# Supplementary material for: Association between Body Mass Index and Renal Outcomes Modified by Chronic Kidney Disease and Anemia: The Obesity Paradox for Renal Outcomes
Source: J Clin Med. 2022 May 15;11(10):2787. doi: 10.3390/jcm11102787 (PMC9144670; doi:10.3390/jcm11102787)
Supplement: Supplementary file 1 [file jcm-11-02787-s001.zip › jcm-1721256-supplementary/50. Supplementary tables/Supplementary Table S1. Multivariate linear regression for waist-to-hip ratio (WHR_ per 0.05 increase).pdf]

Supplementary Table S1. Multivariate linear regression for waist-to-hip ratio (WHR; per 0.05 increase)

	Beta coefficient	95% CI Beta coefficient	P value
Constant	0.734		
Age (years)	0.002	0.001 to 0.002	<0.001
Gender (male vs female)	-0.034	-0.043 to -0.026	<0.001
eGFR (ml/min/1.73 m^2)	0.000	0.000 to 0.000	0.159
Upcr log	0.007	-0.001 to 0.015	0.107
Diabetes mellitus	0.017	0.008 to 0.026	<0.001
Cardiovascular disease	0.007	-0.002 to 0.016	0.144
Glycosylated hemoglobin (%)	0.003	0.000 to 0.006	0.022
Hemoglobin (g/dl)	0.001	0.000 to 0.001	<0.001
Hemoglobin (g/dl)	0.003	0.001 to 0.006	0.004
in CKD 1-3	0.005	0.002 to 0.008	0.002
in CKD 4-5	0.001	-0.003 to 0.005	0.702
Albumin (g/dl)	0.002	-0.007 to 0.010	0.688
Cholesterol log	-0.005	-0.040 to 0.030	0.777
CRP ln	0.006	0.002 to 0.010	0.005
Phosphorus (mg/dl)	-0.003	-0.007 to 0.001	0.177
Uric acid (mg/dl)	0.003	0.001 to 0.005	0.003
Ferritin log	0.000	-0.009 to 0.008	0.918
GPT (U/l)	0.000	0.000 to 0.000	0.239
Bicarbonate (mEq/l)	-0.001	-0.003 to 0.000	0.006

Abbreviations: WHR: waist-to-hip ratio, CI: confidence interval, eGFR: estimated glomerular filtration rate, Upcr: urine protein and creatinine ratio, CKD: chronic kidney disease, CRP: C-reactive protein, GPT: glutamate pyruvate transaminase.
