# Supplementary material for: Association between Body Mass Index and Renal Outcomes Modified by Chronic Kidney Disease and Anemia: The Obesity Paradox for Renal Outcomes
Source: J Clin Med. 2022 May 15;11(10):2787. doi: 10.3390/jcm11102787 (PMC9144670; doi:10.3390/jcm11102787)
Supplement: Supplementary file 1 [file jcm-11-02787-s001.zip › jcm-1721256-supplementary/50. Supplementary tables/Supplementary Table S2. Association between BMI, renal outcomes, and mortality according to Hb levels among patients with CKD stage 4¿C5.pdf]

Supplementary Table S2. Association between BMI, renal outcomes, and mortality according to Hb levels among patients with CKD stage 4–5

		BMI (kg/m^2)					
		G1	G2	G3	G4	G5	G6
		15.0-20.0	20.0-22.5	22.5-25.0	25.0-27.5	27.5-30.0	30.0-35.0
HR for renal outcome							
Hb < 11 g/dl	Unadjusted	1.22 (0.91-1.63)	1.06 (0.81-1.39)	1.12 (0.86-1.45)	1.11 (0.84-1.46)	1.01 (0.75-1.37)	1 (reference)
	Fully-adjusted	1.41 (1.05-1.90)*	1.09 (0.83-1.44)	1.12 (0.86-1.47)	1.09 (0.82-1.44)	1.01 (0.74-1.38)	1 (reference)
Hb ≥ 11 g/dl	Unadjusted	0.94 (0.44-1.99)	1 (reference)	1.04 (0.67-1.61)	1.17 (0.75-1.82)	1.26 (0.79-2.02)	1.41 (0.87-2.29)
	Fully-adjusted	2.54 (1.14-5.63)*	1 (reference)	2.12 (1.29-3.50)*	1.97 (1.19-3.24)*	3.04 (1.79-5.18)**	2.64 (1.53-4.55)**
HR for mortality							
Hb < 11 g/dl	Unadjusted	1.61 (1.03-2.52)*	1.45 (0.94-2.22)	1.33 (0.87-2.02)	1.17 (0.75-1.81)	1.35 (0.84-2.16)	1 (reference)
	Fully-adjusted	1.83 (1.15-2.90)*	1.57 (1.01-2.43)*	1.26 (0.82-1.93)	1.13 (0.72-1.77)	1.14 (0.71-1.84)	1 (reference)
Hb ≥ 11 g/dl	Unadjusted	2.31 (0.75-7.09)	1.45 (0.49-4.34)	1.53 (0.52-4.55)	2.20 (0.74-6.50)	1 (reference)	2.15 (0.58-8.00)
	Fully-adjusted	3.25 (0.95-11.09)	1.37 (0.43-4.39)	1.32 (0.42-4.16)	1.41 (0.44-4.55)	1 (reference)	1.30 (0.31-5.38)

Values expressed as hazard ratio (HR) and 95% confidence interval (CI).
Fully adjusted model: adjusted for age, sex, eGFR, Upcr log, cardiovascular disease, smoking history, cancer, severe liver disease, and hypertension.
*$P < 0.05$ compared with reference BMI category.
**$P < 0.001$ compared with reference BMI category.
Renal outcomes are defined as renal replacement therapy and a 50% decline in eGFR.
Abbreviations: BMI: body mass index, Hb: hemoglobin, CKD: chronic kidney disease, HR: hazard ratio, Upcr: urine protein and creatinine ratio.
