# Supplementary material for: Association between Body Mass Index and Renal Outcomes Modified by Chronic Kidney Disease and Anemia: The Obesity Paradox for Renal Outcomes
Source: J Clin Med. 2022 May 15;11(10):2787. doi: 10.3390/jcm11102787 (PMC9144670; doi:10.3390/jcm11102787)
Supplement: Supplementary file 1 [file jcm-11-02787-s001.zip › jcm-1721256-supplementary/50. Supplementary tables/Supplementary Table S3. Association between WHR, renal outcomes, and mortality according to CKD stage.pdf]

Supplementary Table S3. Association between WHR, renal outcomes, and mortality according to CKD stage

		WHR				
		Q1	Q2	Q3	Q4	Q5
HR for renal outcome						
CKD 1-3	Unadjusted	0.95 (0.65-1.37)	0.89 (0.62-1.28)	1.03 (0.72-1.46)	0.86 (0.60-1.25)	1 (reference)
	Fully-adjusted	1.33 (0.90-1.97)	1.20 (0.82-1.78)	1.26 (0.88-1.82)	1.02 (0.70-1.48)	1 (reference)
CKD 4-5	Unadjusted	1.35 (1.15-1.59)**	1.03 (0.87-1.21)	1 (reference)	0.97 (0.82-1.15)	1.21 (1.02-1.43)*
	Fully-adjusted	1.02 (0.86-1.21)	1.08 (0.91-1.28)	1 (reference)	1.05 (0.89-1.25)	1.16 (0.97-1.38)
HR for mortality						
CKD 1-3	Unadjusted	1.04 (0.69-1.57)	0.66 (0.42-1.04)	0.74 (0.47-1.14)	1 (reference)	1.43 (0.97-2.09)
	Fully-adjusted	1.82 (1.18-2.81)*	1.24 (0.77-1.99)	1.07 (0.68-1.67)	1 (reference)	1.27 (0.86-1.88)
CKD 4-5	Unadjusted	1.22 (0.94-1.57)	1.06 (0.82-1.39)	1 (reference)	1.70 (1.33-2.17)**	2.06 (1.63-2.61)**
	Fully-adjusted	1.41 (1.09-1.83)*	1.21 (0.93-1.58)	1 (reference)	1.56 (1.22-1.99)**	1.40 (1.10-1.78)*

Values expressed as hazard ratio (HR) and 95% confidence interval (CI).
Fully adjusted model: adjusted for age, sex, eGFR, Upcr log, cardiovascular disease, smoking history, cancer, severe liver disease, and hypertension.
[*] $P < 0.05$ compared with reference WHR category.
[**] $P < 0.001$ compared with reference WHR category.
Renal outcomes are defined as renal replacement therapy and a 50% decline in eGFR.
Abbreviations: WHR: waist-to-hip ratio, CKD: chronic kidney disease, HR: hazard ratio, Upcr: urine protein and creatinine ratio.
