# Supplementary material for: Association between Body Mass Index and Renal Outcomes Modified by Chronic Kidney Disease and Anemia: The Obesity Paradox for Renal Outcomes
Source: J Clin Med. 2022 May 15;11(10):2787. doi: 10.3390/jcm11102787 (PMC9144670; doi:10.3390/jcm11102787)
Supplement: Supplementary file 1 [file jcm-11-02787-s001.zip › jcm-1721256-supplementary/50. Supplementary tables/Supplementary Table S4. Association between WHR, renal outcomes, and mortality according to Hb levels.pdf]

Supplementary Table S4. Association between WHR, renal outcomes, and mortality according to Hb levels

		WHR				
		Q1	Q2	Q3	Q4	Q5
HR for renal outcome						
Hb ≥ 11 g/dl	Unadjusted	0.88 (0.64-1.20)	1.15 (0.87-1.52)	1.22 (0.93-1.60)	1 (reference)	1.15 (0.85-1.56)
	Fully-adjusted	1.47 (1.05-2.07)*	1.33 (1.00-1.78)*	1.24 (0.93-1.65)	1 (reference)	1.06 (0.78-1.45)
Hb < 11 g/dl	Unadjusted	1.24 (1.04-1.47)*	1 (reference)	1.01 (0.84-1.22)	0.92 (0.76-1.10)	1.18 (0.98-1.41)
	Fully-adjusted	1.01 (0.84-1.20)	1 (reference)	1.01 (0.83-1.22)	1.04 (0.86-1.26)	1.15 (0.95-1.40)
HR for mortality						
Hb ≥ 11 g/dl	Unadjusted	1.29 (0.85-1.95)	1 (reference)	1.28 (0.87-1.89)	1.89 (1.30-2.73)*	2.60 (1.80-3.75)*
	Fully-adjusted	1.24 (0.80-1.90)	1 (reference)	1.08 (0.72-1.60)	1.29 (0.88-1.88)	1.38 (0.93-2.03)
Hb < 11 g/dl	Unadjusted	1.31 (0.99-1.74)	1.03 (0.76-1.40)	1 (reference)	1.57 (1.19-2.07)*	1.96 (1.50-2.55)*
	Fully-adjusted	1.45 (1.09-1.92)*	1.12 (0.82-1.52)	1 (reference)	1.48 (1.12-1.97)*	1.42 (1.08-1.86)*

Values expressed as hazard ratio (HR) and 95% confidence interval (CI).
Fully adjusted model: adjusted for age, sex, eGFR, Upcr log, cardiovascular disease, smoking history, cancer, severe liver disease, and hypertension.
*$P < 0.05$ compared with reference WHR category.
Renal outcomes are defined as renal replacement therapy and a 50% decline in eGFR.
Abbreviations: WHR: waist-to-hip ratio, Hb: hemoglobin, HR: hazard ratio, Upcr: urine protein and creatinine ratio.
