# Supplementary material for: Association between Body Mass Index and Renal Outcomes Modified by Chronic Kidney Disease and Anemia: The Obesity Paradox for Renal Outcomes
Source: J Clin Med. 2022 May 15;11(10):2787. doi: 10.3390/jcm11102787 (PMC9144670; doi:10.3390/jcm11102787)
Supplement: Supplementary file 1 [file jcm-11-02787-s001.zip › jcm-1721256-supplementary/50. Supplementary tables/Supplementary Table S5 Baseline characteristics of individuals with different CKD stages.pdf]

Supplementary Table S5. Baseline characteristics of individuals with different CKD stages

	CKD stages							*P* value (ANOVA)
	All	CKD stage 1	CKD stage 2	CKD stage 3a	CKD stage 3b	CKD stage 4	CKD stage 5	
No. of patients	3605	111 (3.1%)	231 (6.4%)	408 (11.3%)	766 (21.2%)	940 (26.1%)	1149 (31.9%)	
**Demographics and medical history**								
Body mass index (Kg/m^2)	24.6 (3.7)	24.5 (4.5)	25.2 (3.6)	25.5 (3.3)	24.8 (3.5)	24.8 (3.6)	24.0 (3.7)	0.164
**Laboratory data**								
Hemoglobin (g/dl)	11.2 (2.5)	13.6 (2.0)	13.9 (1.9)	13.5 (1.9)	12.5 (1.9)	10.8 (1.8)	9.1 (1.4)	<0.001
Hemoglobin > 10.0 g/dl	2272 (63.0%)	106 (95.5%)	226 (97.8%)	389 (95.3%)	687 (89.7%)	614 (65.3%)	250 (21.8%)	<0.001
Albumin (g/dl)	3.8 (0.6)	3.9 (0.7)	3.9 (0.6)	4.1 (0.5)	4.0 (0.5)	3.8 (0.5)	3.7 (0.5)	<0.001
Iron (mg/dl)	73.8 (36.0)	79.2 (56.3)	90.1 (57.7)	86.7 (30.5)	79.3 (33.3)	69.9 (29.3)	64.9 (33.0)	<0.001
Ferritin (ng/ml)	276.4 (277.0)	188.3 (279.3)	256.9 (271.9)	236.2 (224.4)	243.2 (230.5)	266.5 (259.7)	333.2 (323.6)	<0.001
Iron saturation (%)	29.1 (15.7)	33.8 (28.7)	36.6 (25.8)	31.4 (13.2)	29.1 (12.9)	28.2 (13.6)	27.2 (14.9)	<0.001
Bicarbonate (mEq/l)	22.1 (4.5)	25.4 (3.9)	25.7 (3.7)	25.1 (3.0)	24.1 (3.3)	22.1 (3.7)	18.5 (3.9)	<0.001
C-reactive protein > 3.0 mg/l	1183 (32.8%)	31 (27.9%)	70 (30.3%)	94 (23.0%)	228 (29.8%)	310 (33.0%)	450 (39.2%)	<0.001

Data are presented as mean (standard error), median (interquartile range), or count (percentage %).

Abbreviations: CKD: chronic kidney disease.
